# Effects of different primers and colouring solutions on orthodontic bonding: shear bond strength and colour change

**DOI:** 10.1007/s00784-024-05665-8

**Published:** 2024-04-25

**Authors:** Hatice Kübra Eren, Fundagül Bilgiç Zortuk

**Affiliations:** Faculty of Dentistry, Department of Orthodontics, Hatay Mustafa Kemal University, Hatay, 31060 Turkey

**Keywords:** Bonding, Colour change, Primer, Solution

## Abstract

**Objectives:**

This in vitro study evaluated the effect of different colouring solutions and primer systems used in the bonding of brackets on enamel colour change and bond strength.

**Materials and methods:**

120 premolar teeth were divided into four main groups; brackets were bonded with 37% orthophosphoric acid + Transbond XT Primer in Group 1, 3 M Single Bond Universal in Group 2, Transbond Plus SEP in Group 3, and G-Premio Bond in Group 4. Each group was divided into three subgroups, and the teeth were placed in a cup containing coffee and tea mixture, in a cup containing cola and in distilled water. A bond strength test was applied to all teeth. Colour measurements of all teeth were performed at 2 different times: before bonding and after the bond strength test.

**Results:**

The average bond strength of the 37% orthophosphoric acid group was higher than that of the other groups. The effect of primer and solution groups on colour change was statistically significant (*p* = 0.001 and *p* = 0.023, respectively).

**Conclusions:**

In this study, the bond strength was clinically sufficient in all primer groups. The highest colour change was observed when the tea–coffee solution and Transbond Plus SEP primer were used.

**Clinical relevance:**

This study has identified enamel discoloration and bond strength from different colouring solutions and primer systems used for bonding braces, which can be used to inform clinicians and patients to achieve better treatment results.

## Introduction

For orthodontic treatment to be successful, the bond strength between the bracket and the tooth must be sufficiently resistant to masticatory forces and must be able to transmit the mechanical forces applied during treatment. The orthodontic adhesive should ensure that the bracket remains bonded to the enamel surface during orthodontic treatment and allow the bracket to be easily removed when necessary without damaging the enamel [1]. It has been reported that the bonding force between the bracket and tooth should be between 6 and 10 MPa to be clinically adequate, but should not exceed 14 MPa to avoid damage to the enamel surface [2].

Modern adhesives are classified under three main groups according to their mechanisms of action and application methods: etch and rinse adhesives, self-etching adhesives and glass ionomer adhesives [3]. An analysis of in vitro and in vivo studies are analysed, it is seen that etch and rinse systems provide a strong bonding force in the enamel [4]. Although the bonding strengths were found to be lower than conventional systems, the bonding strength values of self-etch primers were also reported to be sufficient for clinical use (6–8 MPa) [5]. Although successful in bonding, conventional systems also have disadvantages. It has been reported that when compared with self-etch and resin glass ionomer systems, conventional systems cause the most tooth discoloration [6].

Universal adhesives are named such because they are designed for use with different applications, such as etch and rinse systems, self-etch systems and selective etching [7]. Although universal adhesives have a similar composition to single-step self-etching systems, most universal adhesives also contain specific carboxylate and/or phosphate monomers that ionically bond to calcium in hydroxyapatite. Among these monomers, methacryloyloxydecyl dihydrogen phosphate (10-MDP) is now included in the composition of most universal adhesives and improves bond strength by performing chemical bonding and micromechanical bonding in both enamel and dentin [8].

Undesirable changes, such as enamel surface discoloration, microcracks, abrasion and white spot lesions, can be observed during and after fixed orthodontic treatment [9]. Considering that enamel discoloration is largely caused by residual adhesives that cannot be removed from the enamel prisms by cleaning methods as a result of orthodontic treatment, many researchers have also examined the effect of different etching systems on discoloration [10, 11]. It is also known that resins that cannot penetrate enamel prisms up to 50 μm cause discoloration because they cannot be removed from the tooth surface by debonding and cleaning procedures [9, 12]. Coloured foods and products resulting from the corrosion of metals used in orthodontic treatment also lead to enamel discoloration [13].

When the literature was reviewed, there were no studies investigating the effect of different primer systems and the use of different liquids on both enamel bonding success and the colour changes observed on the enamel surface in orthodontic treatment. In this study, we aimed to compare the effects of different colouring solutions and different primer systems used for bonding braces on enamel discoloration and bond strength in vitro. Our hypothesis is as follows: “The use of different solutions with conventional and self-etch adhesive systems has no differing effects on the bond strength of the brackets and the colour changes observed in the enamel.”

## Materials and methods

Before starting our study, ethical approval was obtained from Clinical Research Ethics Committee (protocol number 2021/06). G Power (3.1.9.7) software was used to determine the sample size for our study. Taking Tavares et al. [14] as an example, the sample size was determined as five in each group at an 80% power level and an effect size of 0.50. Considering the possible loss of samples during the experiments, 10 samples were used for each group. A total of 120 extracted premolar teeth were used in this study.

After extraction, the teeth were washed to remove blood and tissue residues. The extracted teeth were then preserved in 0.1% thymol solution for 3 months in a dark environment to prevent the enamel structure from deteriorating and to prevent bacterial growth. Before starting the experiments, the teeth were kept in distilled water, and the solution was renewed once a week.

For the bond strength test, moulds in the form of rectangular prisms measuring 40 × 20 × 20 mm were prepared. The teeth were placed in these moulds using autopolymerising acrylic. During this process, the acrylic did not touch the crowns of the teeth.

Before colour measurement, all teeth were cleaned with a fluorine-free polishing paste using a soft bristle brush at a low speed for 10 s. The polished teeth were then washed under water for 20 s and dried with an air water spray for 20 s.

**Fig. 1:**
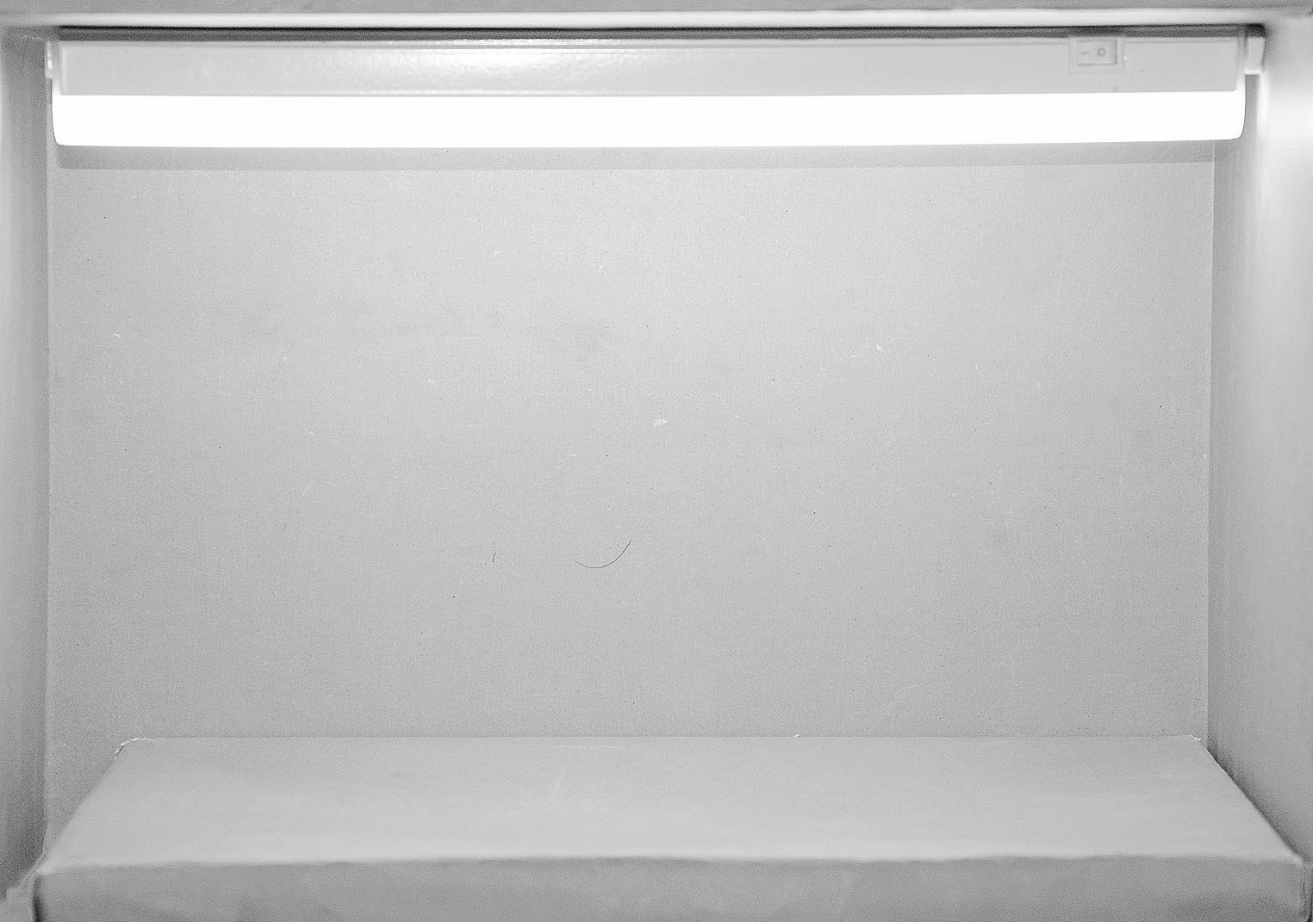
Box for colour measurement

All teeth were measured by a single investigator on the same day and under the same room conditions. The shade measurement was performed in a special shade determination box with an inner surface covered with a neutral grey background (Fig. 1). The box was illuminated with a 6500 K Philips daylight-led bulb that mimics natural daylight, and the teeth were positioned at a 45° angle to the light source. A Vita Easyshade spectrophotometer (Vita Zahnfabrik, H.Rauter GmbH&Co, Germany) was used for colour measurement. The suitable mouthpart of the spectrophotometer’s camera was placed at a 90° angle on the specimen surface. To ensure accurate evaluation, colour measurements were taken from the middle third of the vestibule of all teeth. Each measurement was made three times from the same area of the samples to reduce the margin of error, and the average of the three measurements was calculated. To enforce standardisation, the spectrophotometer device was calibrated after every five measurements in accordance with the manufacturer’s recommendation. All teeth in our study were measured for colour using a Vita Easyshade spectrophotometer at two separate times both before bonding and after bond strength testing.

The CIE (Commission Internationale de l’Eclairage) L*a*b* colour system used in our study is accepted as the international standard for colour identification and is frequently used by many researchers. In this system, absolute measurements are colour evaluated using the axes formed by the coordinates L*, a* and b*, and the colour change is calculated as ∆Eab. The mean values of L, a and b were calculated for each sample, and the ∆Eab value was calculated for each sample using the CIE formula ∆Eab = [[∆L]^2^ + [∆a]^2^ + [∆b]^2^ ] ^1/2^= [[L_2_-L_1_] ^2^ + [a_2_-a_1_] ^2^ + [b_2_-b_1_] ^2^ ] ^½^. In terms of comparing the differences between colour measurements, an ΔE value of zero indicates that the colour is stable, while values below ∆Eab 1 indicate clinically acceptable colour change and these values cannot be perceived by the human eye. ∆Eab value greater than 1 and less than 3.7 define a visually noticeable but clinically acceptable colour change. ∆Eab values greater than 3.7 indicate clinically unacceptable colour change [6, 15]. In our study, a ∆Eab value of 3.7 was accepted as the threshold.

The teeth were divided into four main groups, and bonding was performed using 37% orthophosphoric acid + Transbond XT Primer (3 M Unitek, Monrovia, CA, USA) in Group 1, 3 M Single Bond Universal (3 M Espe, St. Paul, MN, USA) in Group 2, Transbond Plus Self-Etching Primer (SEP), (3 M Unitek, Monrovia, CA, USA) in Group 3, and G-Premio Bond Universal Adhesive, (GC, Tokyo, Japan) in Group 4. In our study, Mini Master series 18 slot metal brackets (American Orthodontics, Sheboygan, NY, USA) were applied to all teeth.

Group 1: 37% of orthophosphoric acid was applied to the buccal surfaces of washed and dried teeth for 30 s. Transbond XT Primer (3 M Unitek, Monrovia, CA, USA) was applied to the tooth surface with a bond brush followed by air jet and curing for 20 s. The brackets were positioned 4 mm from the incisal edge with Transbond XT Light-Cure Adhesive (3 M Unitek, Monrovia, CA, USA). The excess adhesive was removed and curing was performed for 40 s per tooth, 20 s on each proximal face, with Woodpecker LED curing light (Zhengzhou Smile Dental Equipment Co., Ltd. Henan, China).

Group 2: Self-etching primer was applied to the teeth in this group without etch and rinse. On the vestibular surfaces of the cleaned and dried teeth, 3 M Single Bond Universal Adhesive (3 M ESPE, St. Paul, MN, USA) was applied with circular movements for 3 s using an applicator and dried using air spray. Transbond XT Light-Cure Adhesive (3 M Unitek, Monrovia, CA, USA) was applied to the base of the metal bracket and polymerised as in Group 1.

Group 3: Self-etching primer was applied to the teeth in this group without an etch and rinse. Transbond Plus SEP (3 M Unitek, Monrovia, CA, USA) was applied to the vestibular surfaces of the cleaned and dried teeth with circular movements for 3 s. Activation was initiated by pressing the outermost part of the primer and the solution in the first compartment was transferred to the second compartment. Activation was completed after the outermost part was compressed and folded over the second compartment. Afterwards, the solutions mixed with each other passed into the third compartment and made contact with the applicator. The substance coming from the applicator was applied to the tooth surface for at least 3 s. After the application of the SEP on the tooth surface, air was sprayed on the surface for 1–2 s. Transbond XT Light-Cure Adhesive (3 M Unitek, Monrovia, CA, USA) was applied to the base of the metal bracket and polymerised as in Group 1.

Group 4: Self-etching primer was applied to the teeth in this group without an etch and rinse. G-Premio Bond Universal adhesive (GC, Tokyo, Japan) was applied to the vestibular surfaces of the cleaned and dried teeth with circular movements for 3 s using an applicator and dried using air spray. Transbond XT Light-Cure Adhesive (3 M Unitek, Monrovia, CA, USA) was applied to the base of the metal bracket and polymerised as in Group 1.

After bonding, all samples were kept in an oven fixed at 37˚C in distilled water for 24 h. Each group was divided into three subgroups, and the teeth in group A were placed in the container with a coffee (Nescafe Classic, Nestle, Switzerland) and tea (Yellow Tea, Lipton, Turkey) mixture. The teeth in Group B were placed in the container with cola (Coca-Cola, Turkey), and the teeth in Group C were placed in the container with distilled water so that all surfaces were in contact with the liquid. The coffee and tea solution was prepared by immersing 6 g of ground coffee powder was poured in coffee filter and 2 prefabricated doses (2 × 2 g) of tea was prepared by leaving a tea bag in 200 ml of boiling water at 100 °C for 5 min. The storage containers were kept in an oven fixed at 37˚C for 72 h to simulate the intraoral environment.

**Fig. 2:**
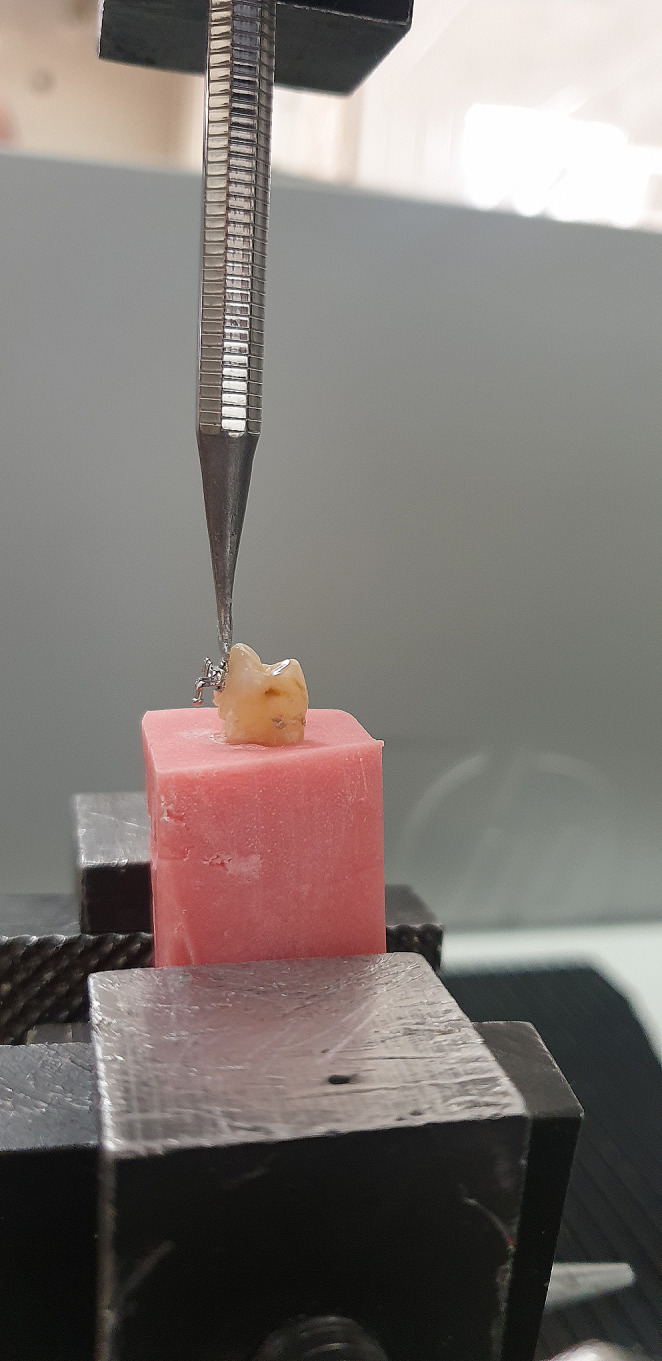
Placing the samples in the instron device

Afterwards, all teeth were subjected to a bond strength test using a Universal test device (Lloyd LRX instruments, Hampshire, England). The prepared specimens were fixed to the device with the force loading end parallel to the tooth surface and close to the bracket base (Fig. 2). Force was applied at a speed of 1 mm/min until the bracket broke away from the tooth surface. With the help of a computer connected to the device, the test results were displayed in Newtons. The numerical values obtained were divided by the bracket base area and calculated in MPa.

**Fig. 3:**
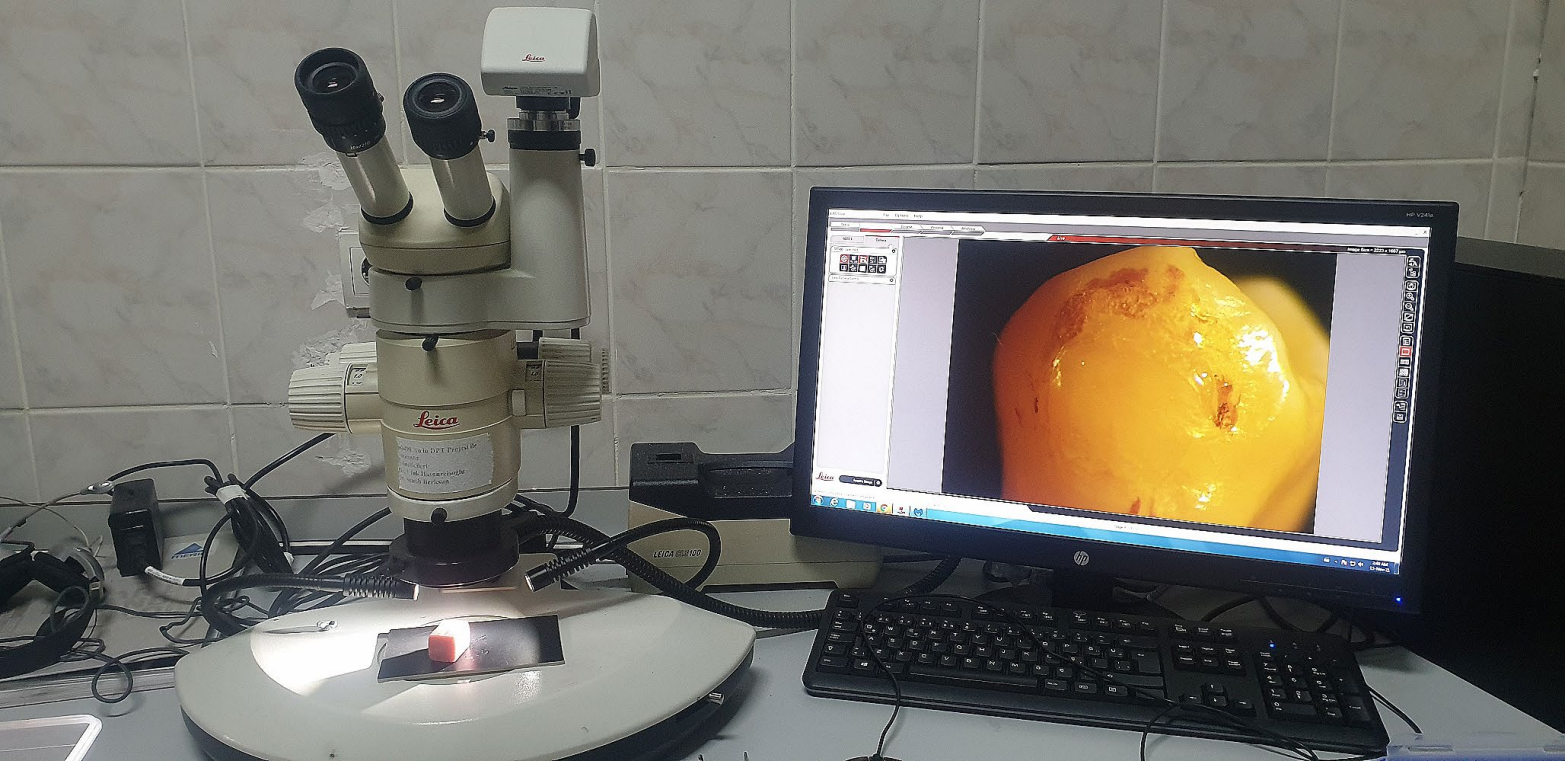
Examination of samples using stereo microscope

After the strength test was performed, the vestibule surface of the teeth was examined and scored by two separate observers using a Leica CLS 100X (Leica Microsystems, Heerbrugg, Switzerland) stereo microscope at 10× magnification (Fig. 3). In our study, the index developed by Bishara and Trulove [16] in 1990 was used to evaluate the amount of adhesive remaining.

The adhesive remnant index (ARI) scale has a range of 1 to 5:

1 = the entire composite, with an impression of the brace base left on the tooth surface.

2 = more than 90% of the composite left over the tooth surface.

3 = more than 10% but less than 90% of the composite left over the tooth surface.

4 = less than 10% of the composite left over the tooth surface.

5 = no composite left over the enamel surface.

The remaining residual adhesive was removed using a 12-blade tungsten carbide bur. After cleaning, polishing was done using a low-speed micromotor with a bristle brush and polishing paste. The polished teeth were then washed for 20 s under running water and dried for 20 s with an air water spray. After these processes, colour measurement was performed again under the same conditions and by the same observer.

### Statistical analysis

The data were analysed with IBM SPSS V23. Conformity to normal distribution was evaluated with the Shapiro–Wilk test. A two-way analysis of variance (ANOVA) was used for the comparison of bond strength and ∆Eab values according to groups and solutions, and multiple comparisons were examined with the Tukey HSD test. Analysis results were presented as mean ± standard deviation and median (minimum – maximum) for the quantitative data. The distribution of ARI scores according to groups was analysed with the Pearson chi-square test. Multiple comparisons were made with the Bonferronni corrected Z test. Interobserver agreement was evaluated with the Kappa test statistic. The analysis results were presented as frequency (percentage). Significance level was presented as *p* < 0.050.

## Results

Among the groups using different primers, the average bond strength of the 37% orthophosphoric acid group was higher than that of the other groups. The average bond strength was 33.5 MPa in the 37% orthophosphoric acid group, 20.57 MPa in the 3 M Single Bond group, 19.9 MPa in the Transbond Plus SEP group and 20.75 MPa in the G-Premio Bond group. There was no significant difference in bond strength between the groups using Transbond Plus SEP, 3 M Single Bond and G-Premio Bond. There was no significant difference in terms of effects between the tea–coffee (21.5 MPa), cola (24.86 MPa) and distilled water (23.53 MPa) solutions on bond strength (Table 1).

**Table 1:** Shear bond strengths (MPa) of the groups

Solutions	Primers				Total Mean ± SD
	Group 1 Mean ± SD	Group 2 Mean ± SD	Group 3 Mean ± SD	Group 4 Mean ± SD	
Tea-Coffee	29,5 ± 13,47	17,8 ± 4,94	20,2 ± 9,26	19,75 ± 3,01	21,5 ± 9,28
Cola	36,25 ± 13,39	22,8 ± 3,85	20,5 ± 12,96	21,5 ± 7,69	24,86 ± 11,56
Distilled water	34,75 ± 13,1	21,1 ± 3,11	19 ± 7,3	21 ± 6,12	23,53 ± 9,79
Total	33,5 ± 13,07^a^	20,57 ± 4,43^b^	19,9 ± 9,79^b^	20,75 ± 5,72^b^	23,3 ± 10,25

SD: Standard deviation;

a-b: In the row, different superscripts indicate statistically significant differences between groups. (*p* > 0.05)

The mean ∆Eab in the Transbond Plus SEP group was higher than that in the 37% orthophosphoric acid and 3 M Single Bond groups. The mean ∆Eab was 6.53 in the 37% orthophosphoric acid group, 6.27 in the 3 M Single Bond group, 8.02 in the Transbond Plus SEP group and 7.45 in the G-Premio Bond group. The mean ∆Eab of the tea and coffee solutions was higher than the mean ∆Eab of distilled water. The mean ∆Eab values were 7.71 for the tea and coffee solution, 6.89 for cola and 6.62 for distilled water (Table 2).

**Table 2:** Mean Commission Internationale de l’Eclairage (CIE) values for all studied teeth according to primers and solutions

Solutions	Primers				Total Mean ± SD
	Group 1 Mean ± SD	Group 2 Mean ± SD	Group 3 Mean ± SD	Group 4 Mean ± SD	
Tea-Coffee	7,16 ± 2,19	7,05 ± 1,8	8,43 ± 1,28	8,19 ± 2,09	7,71 ± 1,87^a^
Cola	5,82 ± 1,38	5,9 ± 1,67	8,17 ± 1,61	7,6 ± 1,24	6,89 ± 1,78^ab^
Distilled water	6,6 ± 1,71	5,85 ± 1,54	7,45 ± 2,29	6,57 ± 1,38	6,62 ± 1,81^b^
Total	6,53 ± 1,8^b^	6,27 ± 1,71^b^	8,02 ± 1,77^a^	7,45 ± 1,68^ab^	7,07 ± 1,86

SD: Standard deviation;

a-b: In the row, different superscripts indicate statistically significant differences between groups. (*p* > 0.05)

A significant difference was found between the distributions for ARI score 4 in terms of the primers (*p* = 0.001). When the distribution of ARI scores was analysed, ARI score 4 with 37.5% was obtained in the 37% orthophosphoric acid group, with 90% was obtained in the 3 M Single Bond group, with 60% was obtained in the Transbond Plus SEP group and the highest rate of score 4 with 87.5% was obtained in the G-Premio Bond group (Table 3).

**Table 3:** Distribution frequency and percentages of adhesive remnant index (ARI) scores according to primers

	Primers				Total	Test Statics	p*
	37% orthophosphoric acid group	3 m single bond	Transbond Plus SEP	G premio bond			
ARI							
2	6 (25)a	0 (0)b	2 (6,7)ab	0 (0)ab	8 (7,4)	28,121	**0,001**
3	7 (29,2)a	2 (6,7)a	7 (23,3)a	2 (8,3)a	18 (16,7)		
4	9 (37,5)a	27 (90)b	18 (60)ac	21 (87,5)bc	75 (69,4)		
5	2 (8,3)a	1 (3,3)a	3 (10)a	1 (4,2)a	7 (6,5)		
Total	24 (100)	30 (100)	30 (100)	24 (100)	108 (100)		

Pearson Chi-Square test results;

a-c: In the row, different superscripts indicate statistically significant differences between groups. (*p* > 0.05)

There was no significant difference between the distributions of the ARI scores according to the solutions (*p* = 0.335). ARI score 2 was highest in cola at 11.1%, ARI score 3 was highest in the tea and coffee solution at 25%, an ARI score 4 was highest in the distilled water at 80.6%, and an ARI score 5 was highest in the tea and coffee solution and cola at 8.3% (Table 4). Statistically significant and high agreement was observed between the two observers in terms of ARI (Ƙ = 0.831; *p* < 0.001) (Table 5).

**Table 4:** Distribution frequency and percentages of adhesive remnant index (ARI) scores according to solutions

		Solutions		Total	Test Statics	p*
	Tea-Coffee	Cola	Distilled water			
ARI						
2	1 (2,8)	4 (11,1)	3 (8,3)	8 (7,4)	6,853	0,335
3	9 (25)	6 (16,7)	3 (8,3)	18 (16,7)		
4	23 (63,9)	23 (63,9)	29 (80,6)	75 (69,4)		
5	3 (8,3)	3 (8,3)	1 (2,8)	7 (6,5)		
Toplam	36 (100)	36 (100)	36 (100)	108 (100)		

Pearson Chi-Square test results;

a-c: In the row, different superscripts indicate statistically significant differences between groups. (*p* > 0.05)

**Table 5:** Evaluation of interobserver agreement in terms of adhesive remnant index (ARI)

	ARI Observer 1				Total	Test Statics	p*
	2	3	4	5			
ARI Observer 2							
2	6 (75)	0 (0)	0 (0)	0 (0)	6 (5,6)	0,831	**< 0,001**
3	2 (25)	16 (88,9)	4 (5,3)	0 (0)	22 (20,4)		
4	0 (0)	2 (11,1)	70 (93,3)	0 (0)	72 (66,7)		
5	0 (0)	0 (0)	1 (1,3)	7 (100)	8 (7,4)		

*Kappa Test results; frequency (percent)

### Stereomicroscope images of the adhesive remnant index

**Fig. 4:**
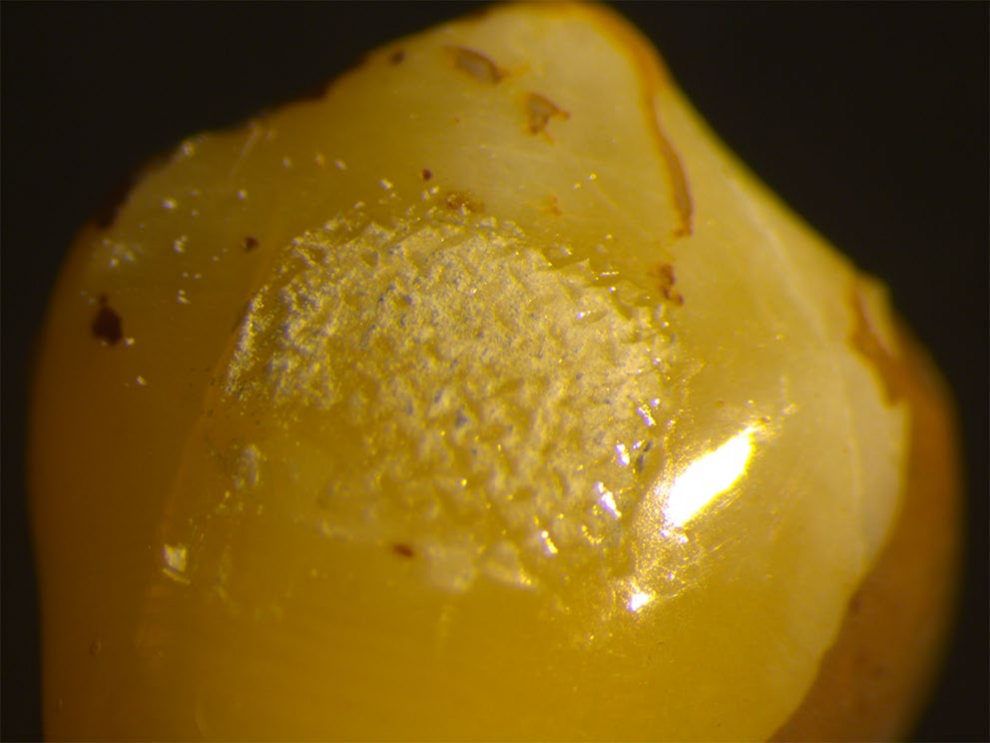
Stereomicroscope image of specimen with an ARI score of 2

**Fig. 5:**
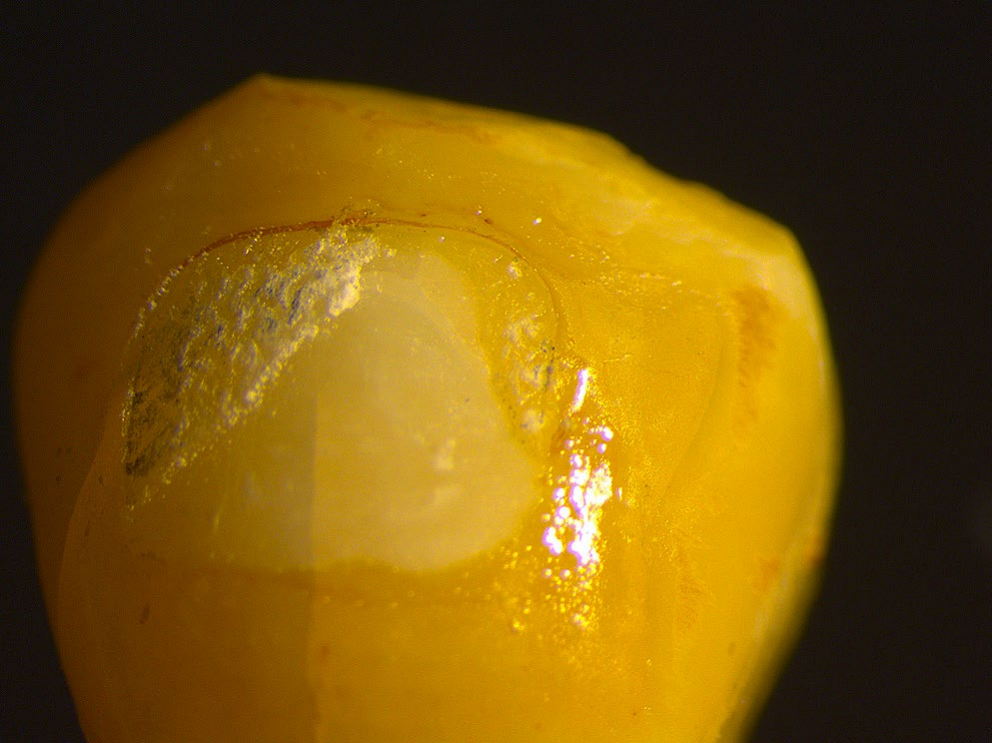
Stereomicroscope image of specimen with an ARI score of 3

**Fig. 6:**
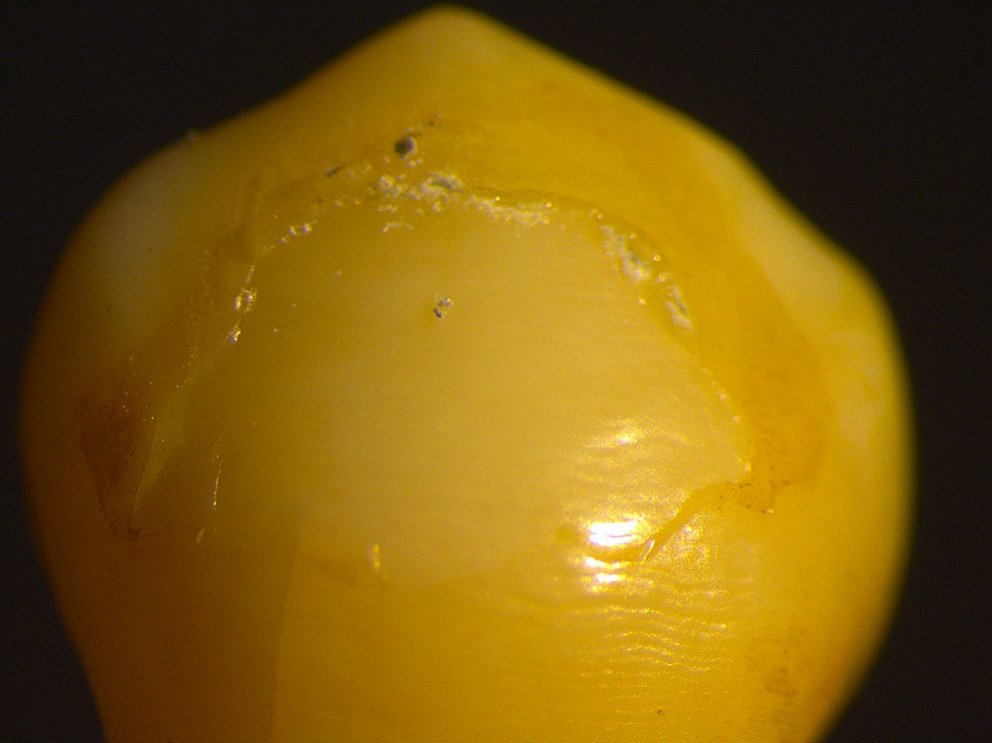
Stereomicroscope image of specimen with an ARI score of 4

**Fig. 7:**
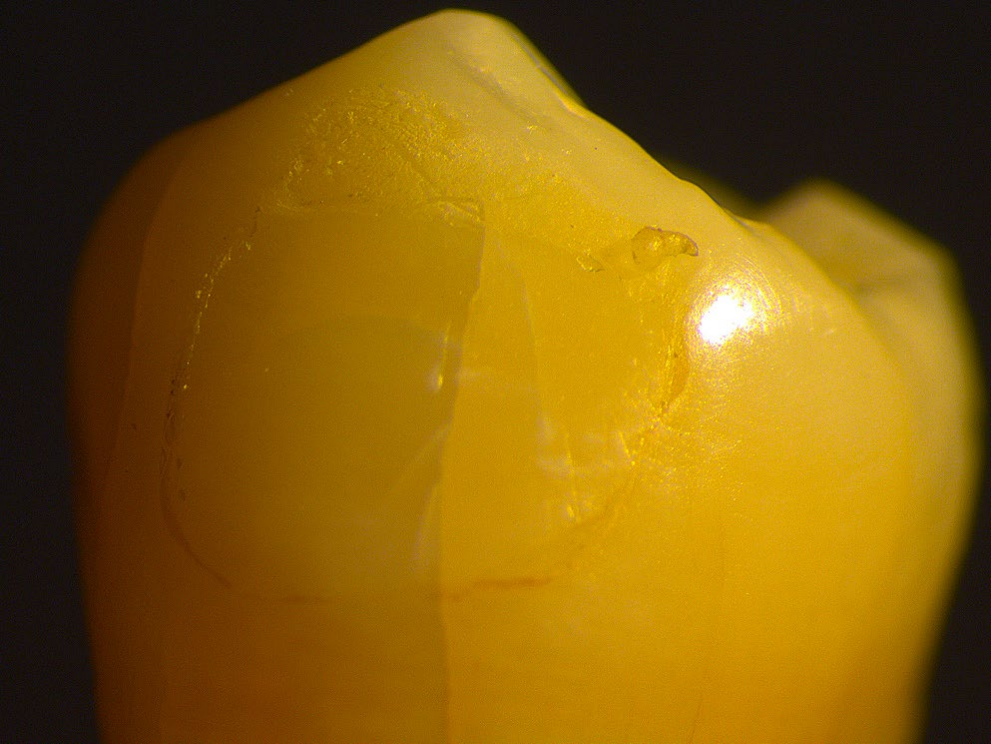
Stereomicroscope image of specimen with an ARI score of 5

In this study, we did not observe any specimens with an ARI score of 1, which means that all the adhesive remained on the tooth surface. For ARI score 2, more than 90% of the adhesive remained on the tooth surface (Fig. 4). For ARI score 3, less than 90% and more than 10% of the adhesive remained on the tooth surface (Fig. 5). For ARI score 4, less than 10% of the adhesive remained on the tooth surface (Fig. 6). For ARI score 5, no adhesive remained on the tooth surface (Fig. 7).

## Discussion

A good orthodontic adhesive should be able to transmit orthodontic forces to the tooth by providing sufficient bond strength during the treatment period, should not damage the enamel surface during the removal phase and should have an aesthetic appearance [5, 6]. In the current study in which we compared four different primer systems including 37% phosphoric acid + Transbond XT Primer, 3 M Single Bond, G-Premio Bond Universal adhesive and Transbond Plus SEP, the mean bond strength of the 37% phosphoric acid + Transbond XT Primer group was significantly higher than that of the other groups. However, the bond strength values of all other groups were recorded as being above clinically acceptable values, and there was no significant difference between them. The average bond strength values were 33.5 ± 13.07 MPa in the group using 37% phosphoric acid + Transbond XT Primer, 20.57 ± 4.43 MPa in the 3 M Single Bond group, 19.9 ± 9.79 MPa in the Transbond Plus SEP group and 20.75 ± 5.72 MPa in the G-Premio Bond Universal adhesive. It was observed that the use of tea–coffee, cola and distilled water was not effective on the bond strength and no significant difference was found between the groups.

Oncag et al. [17] examined the effect of acidic beverages on bond strength in vivo and in vitro. In their study, the lowest bond strength values were observed in both in vivo and in vitro groups using cola, and it was recommended that acidic beverages should not be consumed during orthodontic treatment because they both increased erosion and decreased bond strength. In the present study, unlike Oncag et al.’s [17], it was found that cola and other solutions had no significant effect on bond strength. This difference between the studies may be explained by the application time of the solutions because storage time was 72 h in the current study, while the teeth were exposed to the solution for 3 months in the researchers’ study.

Başaran et al. [5] analysed and compared bond strength using Prompt L-Pop, Futurabond, Transbond Plus SEP and 38% phosphoric acid. Similar to the current study, the bond strength values of the conventional etching method were significantly higher than the other groups, and it was determined that the bond strength in all groups was clinically sufficient.

In a study by Knaup et al. [18] using lower incisors extracted in 2021, self-etching primer systems showed lower bond strength compared to conventional etching systems, but there was no significant difference between them. The bond strength values of all bonding systems were above the minimum 6–10 MPa required for clinical performance [2].

Vaishnav [19] compared the effect of three different primers (Transbond XT, Transbond MIP, Orthofix Anabond) on shear bond strength and found that all the three primers provided an acceptable clinical bond strength.

In the present study, force was applied at a speed of 1 mm/min during the bonding test. It is believed that an increase in blade speed of 1 mm per min provides an average increase of 1.3 MPa in bond strength [20]. In their 2005 study, Bishara et al. [21] reported that decreasing the blade speed from 5 mm/min to 0.5 mm/min increased bond strength by 57%. Bracket fractures occurring in the mouth are believed to be much higher than these speeds [22].

The aim of this study was to investigate the effect of four different primer systems and 3 different solutions on the colour changes that may occur on the enamel surface during orthodontic treatment. According to the CIE L*a*b* colour system used in the present study, the averages of L, a and b values were calculated for each sample and ΔE value was calculated for each sample to compare the differences between colour measurements. As in many other studies, ΔE value of 3.7 was accepted as a threshold value inthe current study, and colour evaluation and discussion of measurements were made based on this threshold value. ΔE value was found to be above 3.7 in all groups in the current study and these values represent clinically unacceptable and visible colour change [15, 23]. Transbond Plus SEP group showed more discoloration than the 37% orthophosphoric acid and 3 M Single Bond groups. There was no significant difference between the G-Premio Bond, 37% orthophosphoric acid and 3 M Single Bond groups in terms of colour change. It is thought that the higher colour change in the Transbond Plus SEP group compared to the other groups could be explained by the fact that the surface residues could not be completely cleaned and that penetration into the enamel prisms was higher.

Zaher et al. [10] compared conventional etching and two different self-etch adhesive methods using extracted premolars and found a significant difference in ∆Eab values between the groups. Self-etch primers provided less resin penetration and therefore caused less iatrogenic discoloration, all other factors being equal. Maatiah et al. [22] compared a 37% phosphoric acid + Transbond XT conventional etching system with a Transbond Plus self-etch adhesive system and found no significant difference between the groups in terms of ΔE.

When previous studies were examined, similar to the current study, the highest colour change was observed in the tea–coffee solution [15, 24, 25]. Cevik et al. [26] found that different liquids (Cherry juice, coffee, Coke, gastric acid, and artificial saliva) caused discolouration on APC flash-free brackets. In the present study, visible colour change was also observed in the cola and distilled water group.

In the present study, Bishara and Trulove’s [16] index was used to evaluate the amount of residual adhesive remaining on the tooth surface after debonding. The amount of residual adhesive remaining on the tooth surface was scored separately by two observers. Statistically significant and high agreement was found between the results of the two observers. When evaluated according to the solutions used, there was no significant difference in the distribution of the ARI scores between the groups.

There are two main views on the interpretation of the residual adhesive remaining on the tooth surface after the brackets are removed. The first of these views is that most of the adhesive remains at the base of the bracket, that is, when the bonding between the bracket base and the adhesive is good, less residual adhesive remains on the enamel surface [27]. In this case, the possibility of fractures and cracks on the enamel surface increased during the debonding phase. In cases where the bond between the enamel surface and the adhesive is high, more residual adhesive remains on the enamel surface. Also there is a possibility of damage to the enamel during removal of the residual adhesive and the time spent at the chair increases in such cases [27]. In the current study, the adhesive did not remain completely on the tooth surface with the trace of the brace in any sample. When the ARI scores were compared, the highest ARI score of 4 was observed in 37.5% of the conventional system group, 90% of the 3 M Single Bond group, 60% of the Transbond Plus SEP group, 87.5% of the G-Premio Bond group and 75% of total samples.

In this study, it was observed that the conventional system group, which was the group with the highest bond strength, contained a higher number of samples with low ARI scores, that is, ARI scores with a higher amount of adhesive remaining on the enamel surface, compared to the other groups.

Kitayama et al. [28] compared the effects of two different conventional systems and two different self-etch primer systems on bond strength. As in the current study, metal brackets were used, and similar ARI scores were found between the groups. Similar to the present study, it was observed that fractures usually occurred at the adhesive–enamel interface. Scougall-Vilchis et al. [29] compared the conventional system and four different self-etch primer systems and found that fractures were mostly observed between the adhesive and the enamel surface. The number of specimens with more residual adhesive remaining on the enamel surface in the conventional system group was higher than in the other groups, similar to the present study.

Kerayechian et al. [30] found that the bracket bonding failure and ARI score were not significantly different between self-etch and conventional acid-etch technique for bonding brackets.

Considering that the oral environment is a complex structure affected by many variables, such as temperature changes, saliva structure, acid level and plaque amount, and that each individual has different nutrition, oral hygiene and chewing habits, it is rather difficult to obtain these conditions with in vitro experiments, and different results can be obtained from in vivo experiments. Another limitation of the current study is the exposure time of the samples in the different beverages. Considering that orthodontic attachments remain in the mouth for an average of 2 years, 72 h of exposure of the teeth to different liquids in this in vitro study can be considered an insufficient time to evaluate discoloration. Therefore, bond strength tests and colour change measurements should be supported by clinical studies.

## Conclusion

Comparisons the effects of different colouring solutions and different primer systems used for bonding braces on enamel discoloration and bond strength yielded the following conclusions:

1. Among the groups in which different primers were used, the average bond strength of the 37% orthophosphoric acid group was higher than that of the other groups, but the bonding strength of all the systems used in the study was above 6–8 MPa, which is considered clinically sufficient.
2. The mean ∆Eab of the Transbond Plus SEP primer group was higher than that of the Transbond XT primer and 3 M Single Bond groups.
3. There was no significant difference between the effects of tea–coffee, cola and distilled water solutions on bond strength.
4. The mean ∆Eab of the tea and coffee solution group was significantly higher than that of distilled water and all the solutions used in the study caused discoloration.
5. Most of the samples of primer groups achieved scores 4, and there were no statistical differences between tea–coffee, cola and distilled water solutions’ scores.
6. The highest colour change was observed when the tea–coffee solution and Transbond Plus SEP primer were used. There was no significant difference between the effects of the other primer groups in terms of colour change.

## Data Availability

No datasets were generated or analysed during the current study.
